# The Retromer Complex and Sorting Nexins in Neurodegenerative Diseases

**DOI:** 10.3389/fnagi.2018.00079

**Published:** 2018-03-26

**Authors:** Hongfeng Zhang, Timothy Huang, Yujuan Hong, Weijie Yang, Xian Zhang, Hong Luo, Huaxi Xu, Xin Wang

**Affiliations:** ^1^Fujian Provincial Key Laboratory of Neurodegenerative Disease and Aging Research, Institute of Neuroscience, College of Medicine, Collaborative Innovation Center for Brain Science, Xiamen University, Xiamen, China; ^2^Neuroscience Initiative, Sanford Burnham Prebys Medical Discovery Institute, San Diego, CA, United States

**Keywords:** the retromer complex, sorting nexin, endosomal sorting, neurodegenerative diseases, Alzheimer’s disease, Parkinson’s disease, Down’s syndrome

## Abstract

The retromer complex and associated sorting nexins (SNXs) comprise a critical trafficking machinery which mediates endosomal protein sorting. Retromer and/or SNX dysfunction has been linked to several neurodegenerative diseases including Alzheimer’s disease (AD), Parkinson’s disease (PD), and Down’s syndrome (DS). In AD, deficiency of the retromer complex or its cargo proteins impairs endosomal trafficking of amyloid precursor protein (APP), resulting in the overproduction of β-amyloid (Aβ). Several SNX components directly interact with APP or APP-cleaving enzymes (β- and γ-secretases) to regulate amyloidogenic APP processing and Aβ generation. In addition, PD-linked mutations in retromer components cause mistrafficking of retromer cargo proteins and mitochondrial dysfunction, and dysregulation retromer-mediated trafficking has been considered as an important cause of hereditary spastic paraplegia (HSP) and neuronal ceroid lipofuscinoses (NCLs). Moreover, SNX27 deficiency is an important contributor for synaptic and cognitive impairment in DS. Here we review recent findings describing the retromer complex and/or SNXs-mediated endosomal sorting in neurodegenerative disorders.

## Introduction

The mammalian retromer complex contains two major subcomplexes: a vacuolar protein sorting-associated protein 26 (VPS26)-VPS29-VPS35 trimeric subcomplex and a membrane-associated sorting nexin (SNX) dimer. VPS35 is the largest retromer component in the core trimer assembly, and forms a scaffold to which VPS26 and VPS29 bind (Seaman, 2005). VPS26 and VPS29 interact with the C-terminal and N-terminal of VPS35, respectively (Shi et al., 2006). In both yeast and mammalian cells, VPS26, VPS29 and VPS35 form a trimer that functions as a “cargo-recognition complex,” which directly recognizes and binds to the cargo molecules. Intracellular transport of numerous transmembrane proteins is regulated by the retromer complex, including VPS10 domain-containing receptors (SORLA, sortilin, and SORCS1-3), cation-independent mannose 6-phosphate receptor (CIM6PR), Wntless and glutamate receptors. Most commonly, the SNX dimer is composed of a combination of SNX1, SNX2, SNX5, and SNX6, all of which possess a SNX-phox-homology (SNX-PX) domain and a C-terminal Bin/Amphiphysin/Rvs (BAR) domain. These SNX-BAR proteins function in different dimeric combinations. Typically, both SNX1 and SNX2 can interact with either SNX5 or SNX6 (van Weering et al., 2012; Sierecki et al., 2014). SNX dimers are capable of inducing membrane remodeling and have a central role in the formation and stabilization of tubules that protrude from endosomes (Frost et al., 2009). SNX-dimer components directly bind to the cargo-recognition complex to form a complete retromer-SNX complex. Recently, the Wiskott–Aldrich syndrome and SCAR homolog (WASH) complex has been found as a newly identified component of the retromer complex (Small and Petsko, 2015). This complex comprises five subunits: CCDC53, WAS protein family homolog 1 (WASH1), KIAA1033, strumpellin, and FAM21. Through interaction between the tail domain of FAM21 and VPS35, the WASH complex can be recruited to endosomal membranes where it promotes actin polymerization and facilitates the efficient sorting of multiple proteins (Seaman et al., 2013).

The retromer complex plays a central role in endosomal trafficking. There are three transport routes originating from endosomes, two of which are mediated by the retromer complex. In retromer-mediated retrograde transport, the retromer complex delivers cargo proteins from endosomes back to the *trans*-Golgi network (TGN). In the retromer-mediated recycling pathway, the retromer complex retrieves and transports endosomal transmembrane proteins directly to the cell surface.

The SNXs belong to a large family of proteins, including ten yeast SNXs and 33 mammalian SNXs (Cullen, 2008). All SNXs contain a Phox homology (PX) domain, which facilitates the interaction of SNXs to phosphatidylinositides (PIs). Most of SNXs localize at early endosomes as they associate with early endosome-enriched phosphatidylinositol 3-phosphate (PI3P). SNXs constitute a diverse group of proteins, as several SNXs contain other functional domains. For example, SNX27 comprises a PDZ protein–protein interaction module that is usually found in proteins enriched within neuronal postsynaptic densities (Wang et al., 2013). SNXs are divided into different subfamilies according to their domain architectures. For example, SNX27 and SNX17 belong to SNX-FERM (4.1/ezrin/radixin/moesin) subfamily as they both contain a FERM domain; SNX1, SNX2, and SNX4 to SNX7 belong to SNX-BAR subfamily as all of them have a BAR domain.

Both postmortem brain tissues and induced pluripotent stem cell (iPSC)-derived neurons from Alzheimer’s disease (AD) patients display enlarged early endosomes (Cataldo et al., 2001; Israel et al., 2012), and neurons from APPswe/PSEN1ΔE9 AD transgenic mice also exhibit multivesicular body (MVB) enlargement (Willen et al., 2017), implicating defects in endosomal trafficking. Intraneuronal accumulation of amyloid precursor protein (APP) fragments, including β-amyloid (Aβ) and β-carboxyl-terminal fragment (β-CTF), contribute to blockage in endosomal trafficking (Small et al., 2017). It has also been suggested that endosomal trafficking defects is an upstream event in AD pathogenesis (Small et al., 2017). Given that the retromer complex and SNXs play critical roles in endocytic trafficking, it will be necessary to investigate whether retromer or SNXs dysfunction contribute to aberrant endocytic trafficking in neurodegeneration. In this review, we summarize the roles of the retromer complex and SNXs in regulating AD associated proteins, such as APP, β-, and γ-secretases. In addition, we review the roles of Parkinson’s disease (PD)-linked *VPS35* mutations in PD-related phenotypes, such as α-synuclein accumulation, DA neuronal loss and locomotor defects. Finally, we summarize the roles of SNX27 and the retromer complex in other neurodegenerative diseases, including Down’s syndrome (DS), hereditary spastic paraplegia (HSP) and neuronal ceroid lipofuscinoses (NCLs).

## The Retromer Complex and SNXs in Neurodegenerative Diseases

### Alzheimer’s Disease (AD)

#### Introduction of AD

**FIGURE 1:**
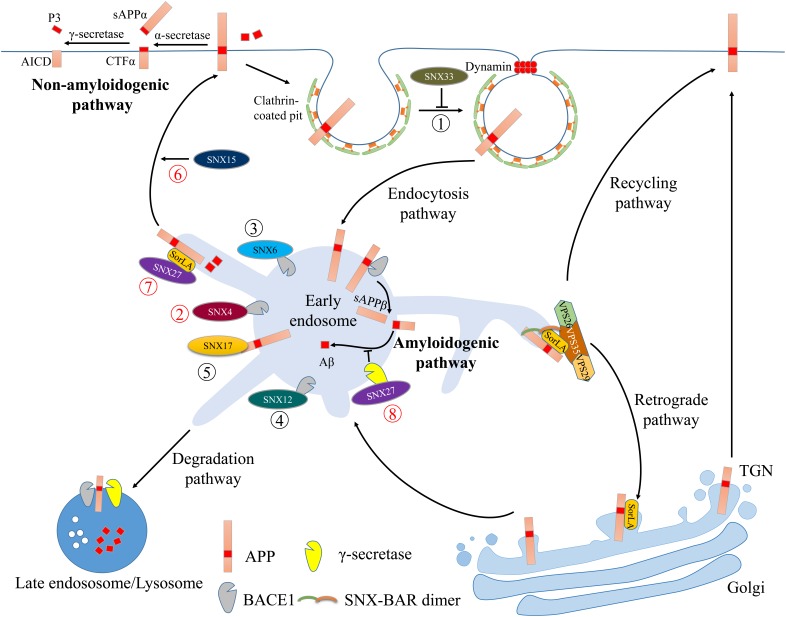
Illustration of the retromer complex and SNXs in regulating AD-related protein trafficking. The pathways and interactions are indicated with colored circled numbers. The novel (recent 3 years) and old interactions were marked with red and black circled numbers, respectively. Cell surface APP undergoes internalization via clathrin- and dynamin-mediated endocytic pathways. Through interactions with dynamin-1, SNX33 inhibits APP endocytosis and prolongs its retention at the cell surface (➀) where non-amyloidogenic APP processing occurs: APP is cleaved first by α-secretase and subsequently by the γ-secretase complex, ultimately generating non-toxic sAPPα, AICD and P3 fragments. Internalized APP can be transported through the early endosome to the late endosome/lysosome for degradation. The acidic endosomal-lysosomal environment enhances β-secretase (BACE1) and γ-secretase to cleave APP and generates Aβ (amyloidogenic pathway). In the early endosome, SNX4, SNX6 or SNX12 can directly interact with BACE1 (➁–➃) and affect its endosomal trafficking, thus modulating amyloidogenic processing. SNX17 can bind to APP and regulate its sorting and processing to Aβ (➄). Both SNX15 and SNX27 promote APP recycling back to the cell surface (➅ and ➆). In addition to interactions with SorLA and APP, SNX27 can also bind to the γ-secretase complex and inhibit the amyloidogenic APP processing (➇). Cooperation between the retromer complex and SorLA can retrieve endosomal APP back to the TGN or to the cell surface, thus trafficking APP away from the intracellular amyloidogenic processing sites.

Alzheimer’s disease is the most common neurodegenerative disorder characterized by memory loss and cognitive impairment. Extracellular neuritic plaques and intracellular neurofibrillary tangles (NFTs) are the two main causes of neuronal dysfunction and degeneration in AD brain (Lee et al., 2001; Zheng and Koo, 2011; Tu et al., 2014). NFTs comprise hyperphosphorylated microtubule-associated protein tau (Gendron and Petrucelli, 2009), while neuritic plaques are produced by Aβ accumulation. Aβ is generated through sequential proteolytic cleavage of APP by two amyloidogenic proteases, namely the β- and γ-secretases (Selkoe, 1998). β-secretase activity is mediated by a type I membrane protein called β-site APP-cleaving enzyme 1 (BACE1), whereas γ-secretase activity is derived from a multi-subunit transmembrane protein complex, including the catalytic presenilin (PSEN) components PS1 and PS2, Nicastrin, presenilin enhancer 2 and APH-1 (Takasugi et al., 2003). Amyloidogenic processing of APP is initiated by BACE1-dependent APP cleavage into a soluble APP fragment (sAPPβ) and membrane-anchored β-CTF. sAPPβ is released into the extracellular space, while β-CTF becomes a direct substrate for γ-cleavage within the lipid bilayer and is subsequently cleaved into the Aβ peptide and the APP intracellular domain (AICD) (“Amyloidogenic pathway” in **Figure 1**). Alternatively, non-amyloidogenic cleavage initiates through α-secretase-dependent APP cleavage within the Aβ region, resulting in the release of a large soluble ectodomain of APP termed sAPPα (“non-amyloidogenic pathway” in **Figure 1**), which exhibits neurotrophic and neuroprotective functions (Edwards et al., 2008).

Interestingly, non-amyloidogenic APP cleavage by α-secretases occurs primarily at the cell surface, whereas amyloidogenic cleavage by BACE1 and the γ-secretase complex occurs in acidified *trans*-Golgi and endosomal environments (Jiang et al., 2014). This suggests that subcellular APP and secretases localization and trafficking influences amyloidogenic Aβ generation. The retromer complex and SNXs-dependent endocytic APP trafficking and processing is depicted in **Figure 1**.

#### The Retromer Complex and AD

It has been reported that retromer components VPS35 and VPS26 are down-regulated in the hippocampus of AD patients (Small et al., 2005), implicating a link between the retromer complex and AD pathogenesis. In a Tg2576 AD mouse model (overexpressing the human KM670/671NL Swedish APP variant), expression of VPS35, VPS26 and retromer cargo proteins CIM6PR and Sortilin related receptor with A-type repeats (SORLA) is also reduced (Chu and Pratico, 2017). Overexpression of VPS35 is observed to attenuate Aβ production in HeLa cells (Small et al., 2005). In contrast, VPS35 depletion using siRNA-targeting strategies elevates Aβ generation (Small et al., 2005; Bhalla et al., 2012); this likely is a result of prolonged APP retention in early endosomes to promote cleavage by BACE1 (Bhalla et al., 2012). *Vps26* deficient mice features elevated Aβ production in the hippocampus, together with impaired hippocampal-dependent memory and synaptic function (Muhammad et al., 2008; Wen et al., 2011). Interestingly, VPS26 deficiency is associated with reductions in VPS35 levels (Muhammad et al., 2008). A *de novo* mutation of VPS35 (VPS35 L625P) was recently identified in an individual with sporadic early-onset AD (Rovelet-Lecrux et al., 2015). This missense variant shows impaired binding to VPS26 and VPS29 (Rovelet-Lecrux et al., 2015), thereby influencing neuronal APP trafficking and Aβ generation.

SORLA, a member of the VPS10 domain receptor family (Andersen et al., 2016), is a well-defined retromer cargo protein. Other members within this family are sortilin, SORCS1, SORCS2, and SORCS3. Genetic studies in humans have identified several late-onset AD-associated mutations in *SORL1*, the gene encoding SORLA (Vardarajan et al., 2015; Nicolas et al., 2016; Verheijen et al., 2016). SORLA expression is reduced in the cortex and hippocampus of AD patients, and shows a strong correlation with cognitive impairment (Scherzer et al., 2004; Sager et al., 2007). Functional studies implicate SORLA as an important modulator for Aβ generation: SORLA overexpression reduces Aβ generation (Andersen et al., 2005; Offe et al., 2006; Rogaeva et al., 2007), while SORLA deletion conversely increases Aβ production (Andersen et al., 2005; Dodson et al., 2008). Mechanistically, SORLA binds to APP and enhances APP delivery to the cell surface from endosomal and Golgi compartments (Andersen et al., 2005), thus trafficking APP away from amyloidogenic processing at the endosome (Fjorback et al., 2012).

Triggering receptor expressed on myeloid cells 2 (TREM2) is an immune-modulatory receptor involved in inflammation and phagocytosis. Several *TREM2* variants are associated with late-onset AD (Guerreiro et al., 2013; Jonsson et al., 2013; Jin et al., 2014). It has been reported that *TREM2* mutations (T66M and Y38C) lead to reduced cell surface delivery and TREM2 degradation (Kleinberger et al., 2014), implicating a role for dysregulated TREM2 trafficking in AD pathogenesis. Interestingly, the retromer complex has been described to facilitate TREM2 trafficking (Lucin et al., 2013): TREM2 can directly interact with VPS35 and VPS35 depletion disrupts TREM2 recycling, leading to the accumulation of TREM2 in lysosomes, thus activating microglia (Yin et al., 2016). Together, these results indicate that dysfunction of the retromer complex results in deficiencies in recycling cargos such as TREM2 to induce microglial defects in AD.

#### SNXs and AD

As mentioned above, endosomal trafficking of APP is regulated by the retromer complex and its cargo proteins (such as SORLA), and deficiency of these proteins is associated with AD pathogenesis. Interestingly, multiple SNXs are also involved in intracellular transport of APP and APP-cleavage secretases (such as BACE1 and α-secretase), thus affecting APP amyloidogenic or non-amyloidogenic pathway. The amyloidogenic or anti-amyloidogenic effect of these SNXs has been summarized in **Table 1**.

**Table 1:** Roles of SNXs in Aβ generation.

SNXs	Amyloidogenic processing	Reference
SNX4	↑	Kim et al., 2017
SNX6	↓	Okada et al., 2010
SNX7	↓	Xu et al., 2018
SNX12	↓	Zhao et al., 2012
SNX15	↓	Feng et al., 2016
SNX17	↓	Lee et al., 2008
SNX27	↓	Wang X. et al., 2014; Huang et al., 2016
SNX33	↓	Schobel et al., 2008


**↑ represent an amyloidogenic effect, ↓ represent an anti-amyloidogenic effect.**

SNX27 exclusively contains a PDZ domain and an additional FERM domain, making it a unique member in the SNX family. SNX27 serves as an early endosome-associated cargo adaptor for endosomal sorting of various transmembrane proteins (Lauffer et al., 2010; Ghai et al., 2013), including β2-adrenergic receptor (Temkin et al., 2011), glucose transporter GLUT1 (Kvainickas et al., 2017) and AD-related proteins, such γ-secretase and APP (Wang X. et al., 2014; Huang et al., 2016). SNX27 interacts with PS1/γ-secretase to inhibit its proteolytic activity, thereby attenuating amyloidogenic Aβ generation (Wang X. et al., 2014) (**Figure 1**). Proteomic analysis indicates that cell surface APP levels are reduced in SNX27-depleted cells, suggesting that SNX27 is required for cell surface APP distribution. As there is no direct interaction between SNX27 and APP (Steinberg et al., 2013), SNX27 regulates APP trafficking likely through an intermediate trafficking component. Recently, SNX27 is shown to bind the cytosolic SORLA tail, forming a ternary complex with APP (Huang et al., 2016). Thus, SNX27 can traffic SORLA and APP to the cell surface to promote non-amyloidogenic sAPPα generation in a SORLA-dependent manner (**Figure 1**) (Huang et al., 2016).

Both SNX27 and SNX17 belong to PX-FERM subfamily. However, unlike SNX27, SNX17 binds directly to the APP cytoplasmic domain via the YXNPXY motif (**Figure 1**). SNX17 depletion decreases steady-state levels of APP with a concomitant increase in Aβ generation (Lee et al., 2008). In addition to controlling APP metabolism, SNX17 also indirectly regulates Aβ generation and clearance. For example, SNX17 binds to low density-lipoprotein receptor 1 (LRP1) and enhances its distribution to the cell surface (van Kerkhof et al., 2005); LRP1 binds to APP and modulates its amyloidogenic processing (Pietrzik et al., 2002, 2004; Waldron et al., 2008), suggesting that SNX17 can also modulate APP processing indirectly through LRP1 trafficking.

In addition, other SNXs including SNX15 and SNX33 are also involved in APP intracellular trafficking. SNX15 accelerates endocytic APP recycling back to cell surface (**Figure 1**) and consequently reduces Aβ production. Overexpression of human SNX15 using adeno-associated virus (AAV) in an APPswe/PSEN1dE9 AD mouse model significantly reduces Aβ deposition in the hippocampus and improves short-term working memory (Feng et al., 2016). Using an expression cloning screen, SNX33 is identified as a novel protein involved in APP endocytosis. Expression of SNX33 in COS cells or HEK293 cells reduces the rate of APP endocytosis in a manner dependent on the endocytic GTPase regulator dynamins (**Figure 1**), thus increasing cell surface APP levels and consequent cleavage by α-secretase (Schobel et al., 2008). A more recent study shows that SNX7 decreases sAPPβ and Aβ production in HEK293T cells possibly by modulating cell surface APP levels (Xu et al., 2018).

Furthermore, several SNXs regulate Aβ production through direct interactions with BACE1. SNX6, a component of the retromer assembly, contains a PX domain and a BAR domain that enable SNX6 to dimerize with SNX1 or SNX2. The heterodimeric subcomplex plays a critical role in the formation and the stabilization of tubules that extend from endosomes. Using a proteomic approach, SNX6 is identified in a BACE1 complex, indicating an interaction between SNX6 and BACE1 (**Figure 1**). *SNX6* knockdown in HEK293 cells enhances BACE1-derived sAPPβ generation, and subsequent Aβ production (Okada et al., 2010). SNX4, which is compositionally similar to SNX6 in the SNX-BAR subfamily, is also capable of interacting with BACE1 (**Figure 1**) and traffics BACE1 away from the degradation pathway, thereby increasing the endosomal BACE1 half-life and consequent Aβ generation (Kim et al., 2017). SNX12 is predominantly expressed in brain tissues and SNX12 levels are significantly decreased in the human AD brains (Zhao et al., 2012), indicating a possible link between SNX12 and AD. Further studies reveal that SNX12 resides in early endosomes (Pons et al., 2012; Priya et al., 2017) and directly interacts with BACE1 (Zhao et al., 2012) (**Figure 1**) to modulate BACE1 endocytosis, thereby affecting APP β-cleavage and Aβ production (Zhao et al., 2012).

### Parkinson’s Disease (PD)

#### Introduction of PD

Parkinson’s disease is the second most prevalent neurodegenerative disease, and affects 1 ∼ 2% of the population over 60–65 years of age (Alves et al., 2008; Massano and Bhatia, 2012). PD is pathologically defined by specific loss of dopaminergic (DA) neurons in the substantia nigra pars compacta and accumulation of α-synuclein-enriched Lewy bodies (Spillantini et al., 1997). Approximately 5 ∼ 10% of PD cases belong to monogenic forms (Lesage and Brice, 2009). Genome-wide association studies (GWAS) have identified multiple mutations in several candidate genes that increase PD susceptibility, including *PINK1*, *DJ-1*, *Parkin*, *SNCA*, *LRRK2*, and *VPS35* (Lill, 2016). As far as we know, genetic relevance of *SNX* genes in PD has not been established. Therefore, in this section, we only discussed the retromer complex component (VPS35) in PD pathogenesis.

#### The Retromer Complex and PD

In 2011, two groups independently identified a missense mutation in VPS35 (VPS D620N) strongly associated with late-onset PD (Vilariño-Güell et al., 2011; Zimprich et al., 2011). Although VPS35 D620N substitution occurs at the binding site of VPS29 (Vilariño-Güell et al., 2011), this mutation does not appear to influence the formation and folding of the retromer’s cargo recognition complex (Zavodszky et al., 2014). Recent studies shows that *Vps35* interacts genetically with the PD-linked gene *parkin* in *Drosophila* (Malik et al., 2015) and VPS35 ubiquitination mediated by Parkin may enhance this interaction (Martinez et al., 2017). These results establish a functional association between the retromer complex and PD. Conventional *Vps35* deletion in mice (*Vps35*^-/-^) results in early embryonic lethality, but the heterozygous animals (*Vps35*^+/-^) are viable (Wen et al., 2011) and display PD relevant neuropathology, including accumulation of α-synuclein, loss of DA neurons, reduction of dopamine, and impairment of locomotor activity (Tang et al., 2015a). Moreover, a DA neuron-specific *Vps35* knockout mouse model exhibits similar deficits at earlier stages (Tang et al., 2015b). Similarly, overexpression of a human VPS35 D620N variant in *Drosophila* also leads to PD-like phenotypes, including loss of tyrosine-hydroxylase (TH)-positive DA neurons, locomotor dysfunction, reduced lifespan and increased sensitivity to rotenone (a PD-associated environmental toxin) (Wang H.S. et al., 2014). In rodents, viral-mediated VPS35 D620N expression or VPS35 D620N knock-in causes dopaminergic neurodegeneration, axonal pathology (Tsika et al., 2014) and deficits in striatal dopamine release (Ishizu et al., 2016).

Mechanistically, VPS35 D620N does not disrupt formation of the VPS26-VPS29-VPS35 heterotrimer (Vilariño-Güell et al., 2011; Follett et al., 2014; McGough et al., 2014; Zavodszky et al., 2014), but impairs transports of multiple retromer cargo proteins. VPS35 D620N decreases its affinity to the endosomal WASH complex which nucleates actin patch formation and endosomal protein sorting (Gomez and Billadeau, 2009; McGough et al., 2014; Zavodszky et al., 2014). Mislocalization of the WASH complex induces defects in the transport of the autophagy-related protein 9A (ATG9A), thereby compromising autophagosome formation and function (Zavodszky et al., 2014), consequently impairing clearance of αsynuclein aggregates. VPS35 D620N also induces impairments in endosome-to-TGN transport of the classical retromer cargo, CIM6PR (MacLeod et al., 2013; McGough et al., 2014). CIM6PR is involved in the delivery of aspartyl protease cathepsin D (Seaman, 2004), which is a lysosomal enzyme responsible for degrading α-synuclein (Sevlever et al., 2008; Cullen et al., 2009). Accordingly, VPS35 D620N disrupts cathepsin D trafficking (Follett et al., 2014), resulting in lysosomal α-synuclein accumulation. Similarly, knockdown of *Vps35* in *Drosophila* perturbes cathepsin D maturation, and induces accumulation of detergent-insoluble α-synuclein in the brain (Miura et al., 2014). Interestingly, a recently study shows that VPS35 depletion or VPS35 D620N overexpression in DA neurons compromises endosome-to-Golgi retrieval of lysosome-associated membrane glycoprotein 2a (LAMP2A), which is critical for α-synuclein degradation (Tang et al., 2015a). Perturbation of the VPS35-LAMP2A-α-synuclein degradation pathway may be responsible for PD pathogenic mechanisms associated with VPS35 deficiency. In addition, VPS35 also regulates trafficking of neurotransmitter receptors such as AMPA and dopamine receptors. VPS35 depletion at mature hippocampal synapses blocks LTP by inhibiting AMPA receptor insertion at the postsynaptic membrane during LTP stimulation; VPS35 D620N re-expression fails to rescue LTP deficits with VPS35 depletion (Temkin et al., 2017). This indicates a possible mechanism underlying cognitive impairment in PD patients carrying pathogenic retromer mutations. Dopamine, an essential neurotransmitter in the brain, regulates various physiological functions, including locomotion, emotion, and behavior. *In vitro* studies demonstrate that VPS35 promotes cell surface delivery of dopamine receptor D1 (DRD1) and therapy enhances DRD1-mediated dopamine signaling, which is compromised by VPS35 D620N expression (Wang C. et al., 2016). Therefore, impaired dopamine signaling observed in PD (Narayanan et al., 2013) can be partially explained by the loss-of-function of VPS35 D620N mutation. We summarized these findings in **Table 2**.

**Table 2:** Abnormalities caused by VPS35 D620N mutation.

Cellular functions	Cargo molecules	Reference
Autophagosome formation	ATG9A	Zavodszky et al., 2014
Mitochondrial dynamics	DLP1, MFN2	Tang et al., 2015b; Wang W. et al., 2016
Endosome to TGN transport	LAMP2A, CIM6PR	MacLeod et al., 2013; McGough et al., 2014; Tang et al., 2015a
Lysosomal degradation	Cathepsin D	Follett et al., 2014
Neurotransmitter signaling	AMPA receptor, DRD1	Wang C. et al., 2016; Temkin et al., 2017


Several neurodegenerative diseases feature mitochondrial dysfunctions in specific neural cell types. For example, aberrations in mitochondrial dynamics and mitophagy have been observed in PD pathogenesis (Bose and Beal, 2016; Gao et al., 2017). A recent study shows that PD-associated VPS35 D620N mutation leads to increased mitochondrial fragmentation and dysfunction, resulting in neuronal loss both *in vitro* and *in vivo* (Wang W. et al., 2016). These aberrations are rescued by inhibiting mitochondrial fission, suggesting excessive mitochondrial fission can induce mitochondrial and neuronal deficits. Mechanistically, the VPS35 D620N mutation enhances interactions between VPS35 and Dynamin-1-Like Protein (DLP1), a key regulator for mitochondrial fission (Chan, 2012), and increases mitochondrial retromer-dependent DLP1 turnover (Wang W. et al., 2016). Thus, retromer defects can perturb mitochondrial homeostasis in favor of excessive mitochondrial fission, leading to mitochondrial dysfunction and neuronal death (Wang W. et al., 2016). Similarly, a more recent study in fibroblasts from a PD VPS35 D620N carrier shows that this mutation leads to increased mitochondrial fission and functional deficits, likely through attenuated mitochondrial respiratory complex I and II, and supercomplex assembly (Zhou et al., 2017). Further, VPS35 deficiency or D620N mutation increases mitochondrial fragmentation and DA neuron death through mitochondrial E3 ubiquitin ligase 1 (MUL1)-mediated degradation of mitofusin 2 (MFN2), a mitochondrial outer membrane protein essential for mitochondrial fusion, thereby impairing mitochondrial fusion (Tang et al., 2015b). In addition, mitochondria in VPS35 D620N overexpressed N27 cells (a dopaminergic cell line) or *Drosophila* models appear to be more vulnerable to mitochondrial stressors, such as 1-methyl-4-phenylpyridinium (MPP^+^) (Bi et al., 2013) and rotenone (Wang H.S. et al., 2014).

Compared to VPS35 D620N, PD-associated R524W mutation is rare, with milder pathogenicity. Results from a recent study suggest that R524W confers loss-of-function through attenuated endosomal recruitment and impaired association with retromer-dependent interacting proteins, including FAM21, Rab7a, and TBC1D5, and perturbs retrograde trafficking of retromer cargo CIM6PR (Follett et al., 2016). Similar to VPS35 D620N, expression of R524W in SH-SY5Y cells increases α-synuclein aggregation compared to wild-type VPS35 (Follett et al., 2016). This suggests a pathological role for VPS35 R524W in PD pathogenesis.

### Down’s Syndrome (DS)

#### Introduction of DS

Down’s syndrome is the most common chromosomal triplication disorder, manifesting in developmental retardation and intellectual disability. Triplication of chromosome 21 in DS leads to overexpression of multiple genes and non-coding RNAs that contributes to DS pathogenesis. All DS patients develop AD pathology in their 40s, amyloid plaques and tau tangles have also been found in DS brains. This is probably due to the presence of *APP* gene that resides on chromosome 21. The extra copy of *APP* gene in DS leads to elevated APP expression and Aβ overproduction (Ness et al., 2012). Recently studies indicate that dysregulated SNXs-mediated endosomal trafficking is implicated in DS neuropathology, thereby contributing to synaptic dysfunction and intellectual impairment.

#### SNXs and DS

SNX27 is enriched in brain, and has been implicated as a major contributor to intellectual impairment in DS (Wang et al., 2013). SNX27 expression is downregulated in human DS brains and in Ts65Dn mouse (an animal model of DS) brains. AAV-mediated SNX27 overexpression in Ts65Dn hippocampus rescues cognitive and synaptic deficits (Wang et al., 2013). Mechanistically, interaction between the SNX27 PDZ domain and glutamate receptor targets (NMDA and AMPA receptors) is shown to enhance their recycling to the plasma membrane. SNX27 depletion reduces glutamate receptor expression in synaptosomal membranes and PSD fractions. Conversely, overexpression of SNX27 leads to increased cell surface distribution of GluA1 (glutamate ionotropic receptor AMPA type subunit 1) and GluN1 (glutamate ionotropic receptor NMDA type subunit 1) (Wang et al., 2013). SNX27 expression has been found to be regulated by CCAAT/enhancer binding protein β (C/EBPβ), a transcription factor downregulated in DS brains through transcriptional targeting by miR-155 encoded on triplicated chromosome 21 (Wang et al., 2013). Taken together, dysregulation of a microRNA-155-C/EBPβ-SNX27 pathway contributes to synaptic and cognitive impairment in DS brains.

In addition to regulating glutamate receptor trafficking, SNX27 deficiency is also a contributor of AD-like pathology in DS brains through regulating Aβ generation. It has been reported that SNX27 depletion enhances Aβ production through modulating (γ-secretase activity (Wang X. et al., 2014) and APP recycling (Huang et al., 2016). Therefore, SNX27 deficiency, at least in part, contributes to Aβ accumulation and AD-like pathology in DS brains (Wang et al., 2013).

Human studies have shown that development and maturation of the white matter correlates with increased motor skills and cognitive function (Paus et al., 1999; Nagy et al., 2004; Schmithorst et al., 2005). Both DS patients and Ts65Dn mice exhibit brain white matter abnormalities, characterized by fewer myelinated axons, thinner myelin sheaths and depleted nodes of Ranvier in the corpus callosum. These abnormalities contribute to deficits in neuronal action potential transmission and cognitive function, which may result from aberrations in oligodendrocyte differentiation and maturation (Olmos-Serrano et al., 2016). A recent study shows that SNX27 modulates oligodendrocyte differentiation through interactions with the myelination-related protein, GRP17 (Gadd related protein, 17 kDa) to mediate cell surface distribution and stability (Meraviglia et al., 2016). These studies indicate that SNX27 dysfunction is associated with brain white matter defects and cognitive deficits in DS through perturbations in oligodendrocyte development, providing additional evidence for the involvement of SNX27 in DS neuropathogenesis.

In addition to SNX27, some retromer components may also be associated with DS. Down syndrome critical region 3 (*DSCR3*), a gene located in human chromosome 21, is upregulated in adult DS brain (Lockstone et al., 2007). DSCR3 is presumed to be a VPS26 paralog and the third member of VPS26 family (in addition to VPS26A and VPS26B). DSCR3, C16orf62 (chromosome 16 open reading frame 62) and VPS29 form a heterocomplex called “retriever.” The retriever complex, together with the WASH complex, regulates SNX17-mediated recycling of α_5_β_1_ integrin (McNally et al., 2017). These findings suggests a potentially important association of retromer/retriever dysfunction in DS pathogenesis.

### Other Neurodegenerative Diseases

Hereditary spastic paraplegia (HSP) is a group of inherited neurodegenerative diseases characterized by progressive spasticity and weakness of the lower limbs (Blackstone et al., 2011). The common pathological feature is the distal axonal degeneration of the corticospinal tract. HSP is a highly heterogeneous disorder with more than 70 identified genotypes up to now (de Souza et al., 2017). Interestingly, multiple proteins encoded by spastic paraplegia genes (*SPGs*) are involved in membrane trafficking and organelle shaping, such as atlastin-1 (encoded by *SPG3A*), spastin (encoded by *SPG4*) and strumpellin (encoded by *SPG8*) (Valdmanis et al., 2007; Freeman et al., 2013; Lo Giudice et al., 2014). As mentioned above, strumpellin is one of the core components of the WASH complex, and the latter is recruited to endosomes by the retromer complex and functions in actin patch formation to facilitate protein trafficking. Strumpellin is required for correct subcellular localization of transmembrane proteins, such as β-2-adrenergic receptor (Freeman et al., 2013). Cells lacking strumpellin display increased endosomal tubulation (Harbour et al., 2010) and enlarged lysosomes (Allison et al., 2017).

Neuronal ceroid lipofuscinoses belong to a group of lysosomal storage disorders (LSDs) characterized by progressive vision loss, seizures, cerebellar atrophy, cognitive impairments and premature death (Nita et al., 2016). The cytopathological hallmark of NCLs is lysosomal accumulation of autofluorescent ceroid lipopigments (Jalanko and Braulke, 2009). Up to now, at least 14 genes mutations have been reported in NCLs, from *CLN1* to *CLN14* (Nita et al., 2016). Among products these genes, CLN5 are shown to interact with CLN2 and CLN3 (Vesa et al., 2002) and reside in endosomal-lysosomal compartment (Isosomppi et al., 2002). CLN5 controls endosome-to-Golgi trafficking of lysosomal sorting receptors (CIM6PR and sortilin) through recruiting the retromer complex to endosomes (Mamo et al., 2012). Upon CLN5 depletion, Hela cells display defects in endosomal recruitment of retromer, which results in degradation of CIM6PR and sortilin (Mamo et al., 2012). In addition, Cathepsin D, a NCLs-associated gene (*CLN10*) product, whose endosomal trafficking is mediated by the retromer complex (Jalanko and Braulke, 2009). Together, these studies implicate involvement of the retromer complex in NCLs pathogenesis.

## Concluding Remarks and Future Perspectives

As discussed above, dysfunction of retromer-mediated trafficking is implicated in the pathogenesis of several neurodegenerative diseases, including AD and PD. Enhancing retromer function could be a good potential therapeutic strategy to reverse endocytic deficits (Berman et al., 2015). In addition, a new class of small molecule pharmacological chaperones that stabilize interactions between members of the retromer complex has been shown to enhance retromer function. Intriguingly, chaperone treatment in cultured hippocampal neurons results in elevated retromer levels and attenuates APP endosomal distribution, thus reducing amyloidogenic APP processing (Mecozzi et al., 2014). Although these findings provide unique insight into potential therapies for AD through enhancements in retromer function, further study will be necessary to thoroughly evaluate these therapeutic effects *in vivo*, and assess safety issues associated with retromer stabilization. Mutations in *LRRK2* are the predominant causal component in familial PD (Roosen and Cookson, 2016). In flies expressing pathogenic *LRRK2* variants, wild-type *VPS35* can extend their lifespan and reverse locomotor deficits (MacLeod et al., 2013; Linhart et al., 2014). Moreover, lentivirus-mediated human *VPS35* overexpression in α-synuclein transgenic mice also reverses α-synuclein induced neurodegeneration (Dhungel et al., 2015). These findings suggest that enhancing VPS35 level may confer neuroprotection in certain PD populations. Therefore, retromer-enhancing compounds may also have significant therapeutic potential in PD.

As reviewed above, several SNX family members including SNX4, SNX6, SNX12, SNX15, SNX17, SNX27, and SNX33 have been identified as trafficking regulators of AD-relevant components, such as APP and BACE1. However, it remains unknown whether other SNXs have similar roles. A comprehensive study of SNXs in AD-related trafficking function will enhance our understanding of AD pathogenesis.

The retromer complex and SNXs are important endosomal trafficking regulators, and their specific functions are embodied mainly through interacting with and sorting transmembrane proteins. For example, SNX27 regulates synaptic and cognitive functions via binding to glutamate receptors. SNX4 and SNX6 affect Aβ generation by directly interacting with BACE1. VPS35 D620N influences mitochondrial dynamics through disrupting association between VPS35 and DLP1. Therefore, searching for novel cargo molecules of SNXs and the retromer complex will contribute to our comprehensive understanding of their roles in biological processes.

In this review, we summarized the functions of the retromer complex and SNXs in five neurodegenerative disorders, including AD, PD, DS, HSP, and NCLs. The retromer complex appears to be implicated in the pathogenesis of all the five diseases, especially important in PD pathogenesis. However, dysfunction of SNXs is more relevant to AD and DS. Considering functional similarity of SNXs and the retromer complex in endosomal soring, we hypothesize that SNXs could also play a role in the pathogenesis of other neurodevelopmental and neurodegenerative diseases, which will be carefully scrutinized in the future.

## Author Contributions

HZ and XW wrote the manuscript. TH, YH, WY, XZ, HL, and HX provided editorial help. All authors read and approved the final manuscript.

## Conflict of Interest Statement

The authors declare that the research was conducted in the absence of any commercial or financial relationships that could be construed as a potential conflict of interest. The reviewer W-CX and handling Editor declared their shared affiliation.
